# A Comparative Analysis of Hydroxyurea Treatment on Coagulation Profile Among Sickle Cell Anaemia Children in Lagos, Nigeria

**DOI:** 10.1155/ah/5002373

**Published:** 2024-11-23

**Authors:** Blessing E. Kene-Udemezue, Abideen O. Salako, Adeseye M. Akinsete, Oluwatosin O. Odubela, Titilope A. Adeyemo

**Affiliations:** ^1^Department of Paediatrics, Lagos University Teaching Hospital, Idi Araba, Lagos, Nigeria; ^2^Clinical Sciences Department, Nigerian Institute of Medical Research, Yaba, Lagos, Nigeria; ^3^Global Child Health, Graduate School of Biomedical Sciences, Global Paediatrics Medicine, St Jude Research Hospital, Memphis, Tennessee, USA; ^4^Department of Paediatrics, Lagos State of Haematology, Blood and Transfusion, Lagos University Teaching Hospital, Lagos, Nigeria

**Keywords:** D-dimer, hydroxyurea, sickle cell anaemia, thrombin antithrombin complex

## Abstract

**Background:** Hydroxyurea (HU) is a disease-modifying therapy with significant clinical and laboratory efficacy among individuals living with sickle cell anaemia (SCA). This is evident through increased fetal haemoglobin, higher packed cell volume, improved red cell hydration, reduced leukocytes, and platelet function. The effect on the coagulation pathway and pathophysiologic mechanism remains unclear, especially in children living with SCA. This study evaluated the coagulation profile using D-dimer and thrombin antithrombin complex (TAT) in children with SCA.

**Methods:** The cross-sectional study was conducted over three months at LUTH among 80 children living with SCA in steady state aged 2–18 years (40 HU exposed and 40 HU naïve, respectively). Blood samples were assayed for D-dimer, TAT, and complete blood count. Descriptive analysis such as mean and standard deviation for normally distributed variables or median and interquartile range for skewed data were used to summarize continuous variables, while proportion or percentages for categorical variables. Univariate analysis and bivariate analysis were done and statistical significance was set at *p* < 0.05.

**Results:** The mean age (±SD) of study participants in both groups was 11.35 (±4.6 years). D-dimer levels (23.27 ng/mL) and TAT (29.79 pg/mL) were significantly lower among HU exposed compared to HU naïve children (62.73 ng/mL and 109.34 pg/mL, respectively) *p* < 0.001. There was a negative correlation between D-dimer and TAT with the duration of HU use (*r* = −0.499, *p*=0.001, and *r* = −0.401, *p*=0.010), respectively. There was a positive correlation between D-dimer and TAT with total WBC (*r* = 0.368, *p*=0.019, and *r* = 0.385, *p*=0.014, respectively) among the HU naïve participants and a negative correlation between D-dimer and TAT with haemoglobin level (*r* = −0.303, *p*=0.047, and *r* = −0.311, *p*=0.041, respectively) among HU exposed children.

**Conclusion:** HU modulates the D-dimer and TAT levels of children living with SCA toward the normal reference range, thus reducing the risk of hypercoagulability and associated sequelae. Therefore, continuous advocacy for HU use should entail close monitoring of adverse effects.

## 1. Introduction

Sickle cell disorder is the most common inherited haemoglobin (Hb) disorder globally with a worldwide distribution [1, 2]. The disease is characterized by acute and chronic end-organ manifestations whose pathophysiologic events are not limited to chronic haemolysis and vaso-occlusion [3, 4]. Hypercoagulability, explained by the alteration in the homeostatic balance between procoagulant and anticoagulant state has also been identified among other evolving pathobiology pathways [4]. Studies have demonstrated the relationship between coagulation activation and the various clinical manifestations of sickle cell disease (SCD) [5–8]. The background hypercoagulable state in children living with SCD has been shown to increase the risk of thromboembolic events [9, 10] such as venous thromboembolism (VTE), pulmonary embolism (PE), and deep vein thrombosis (DVT) [11–13]. These thromboembolic events could have a detrimental impact on the quality of life and overall well-being of the population.

Hydroxyurea (HU) has been instrumental in improving the outcome of people with SCD by increasing fetal Hb (HbF) and red cell hydration while reducing leukocyte and platelet counts [14, 15]. However, the mechanism of action of HU on the coagulation pathway remains unclear.

Few studies have evaluated the effect of HU on coagulation in children with sickle cell anaemia (SCA), using prothrombin time (PT), partial thromboplastin time (PTTK), and other tests that are not as sensitive or specific [16, 17]. The above markers imply a predisposition to abnormal bleeding rather than thrombosis and therefore may not confirm hypercoagulability on their own. This will be reliably determined by direct measurement of markers for fibrinolysis and thrombin generation such as D-dimer and thrombin antithrombin complex (TAT) [18]. These markers have the potential to evaluate the risk of bleeding and thrombosis [19]. This study evaluated the coagulation profile of children with Hb SS genotype (HbSS) on HU using TAT and D-dimer in comparison with their HU naïve counterparts. It is hoped that the findings from this research will contribute to the body of knowledge and promote a better understanding of the relationship between HU and coagulation profile.

## 2. Methods

This comparative cross-sectional study involved children living with SCA aged 2–18 years in steady state who attended follow-up outpatient clinic of the Paediatric department at the Lagos University Teaching Hospital (LUTH), Idi-Araba, Lagos, between February and April 2019. A steady state is defined as persons living with SCA free from pain, infection, or other acute illness for at least four consecutive weeks and having had no blood transfusion during the preceding three to 4 months [20, 21]. The study participants were age and sex-matched children living with SCA on HU and HU naïve. All children living with SCA with confirmed chronic diseases (CVA, CKD, Chronic heart diseases) and blood transfusions in the preceding 3 months were excluded. Ethical approval was obtained from the LUTH Health Research and Ethics Committee [LUTH HREC] (IRB number: 19/12/2008a). Consent and assent were obtained from parents/caregivers and children (aged 7 years and above), respectively, following adequate communication of the study.

At enrollment, a pretested questionnaire was administered to study participants to collect information on their sociodemographic characteristics, regularity of clinic attendance, routine drug therapy, use of HU, and duration of HU use. Thereafter, a detailed clinical examination was carried out and documented in the case report form.

Laboratory procedures: Five milliliters of venous blood was drawn aseptically from each participant's vein into an EDTA (ethylenediamine tetra-acetic acid) tube and used for complete blood counts (CBC), D-dimer, and TAT.

Data analysis: All data collected was cleaned and analyzed using IBM Statistical Package for Social Science (SPSS) version 26 software. Descriptive and inferential statistics were applied during the analysis. Descriptive analysis such as mean and standard deviation for normally distributed variables or median and interquartile range for skewed data were used to summarize continuous variables, while proportion or percentages for categorical variables. Univariate analysis and bivariate analysis were done. A *p* value of less than or equal to 0.05 was set as the level of statistical significance.

## 3. Results

**Figure 1:**
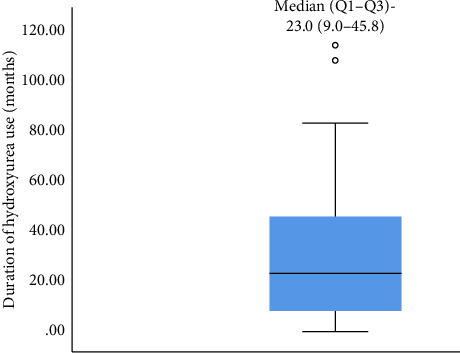
Duration of hydroxyurea use among participants.

A total of eighty (80) children living with SCA were enrolled in the study (40 age and sex-matched HU exposed and HU naïve, respectively). The mean age (±SD) of study participants in both groups was similar at 11.35 (±4.6 years) with a male-to-female ratio of 1:0.9. Most of the study participants (in both arms) were from middle and lower socio-economic classes. There was no significant difference in the demographics between the two groups Table 1. The median (IQR) duration of HU use among the children on HU was 23 (9.0–45.8) months.  Figure 1.

**Figure 2:**
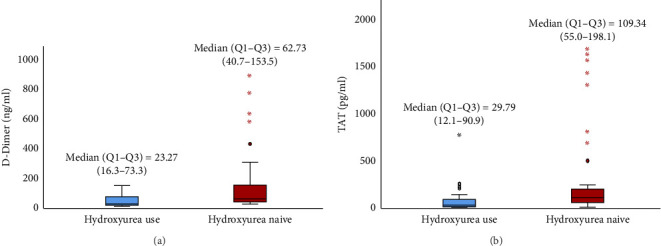
(a) Box plot comparing median D-dimer among participants using hydroxyurea and hydroxyurea naïve. Mann–Whitney *U* value = −3.984, *p* < 0.001^∗^. (b) Box plot comparing median thrombin antithrombin complex among participants using hydroxyurea and hydroxyurea naïve. Mann–Whitney *U* value = −4.23, *p* < 0.001^∗^.

The median (IQR) D-dimer (23.27 (16.3–73.3) ng/mL) and TAT (29.79 (12.1–90.9) pg/mL) in children receiving HU was significantly lower than in HU naïve [62.73 (40.7–153.5) ng/mL and 109.34 (55.0–198.1) pg/mL], (*p* < 0.05) Figures 2(a) and 2(b). The median (IQR) levels of D-dimer and TAT for participants who had used HU for at least 12 months and above were 20.10 (15.7–25.0) ng/mL and 17.90 (10.6–32.0) pg/mL respectively, which were significantly lower than those who were on HU for less than 12 months, (*p* < 0.05) Table 2.

**Figure 3:**
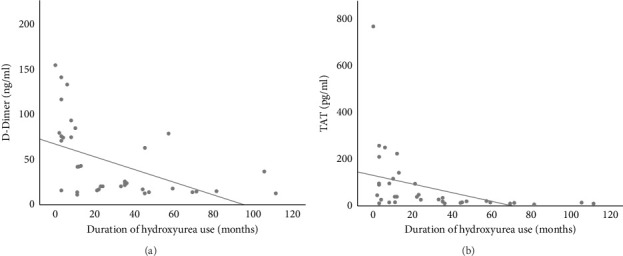
(a) Correlation between duration of hydroxyurea use and D-dimer. Spearman correlation coefficient (*r*) = −0.499, *p*=0.001^∗^. (b) Correlation between duration of hydroxyurea use and TAT. Spearman correlation coefficient (*r*) = −0.401, *p*=0.010^∗^.

Linear regression analysis using Spearman correlation demonstrated a moderate negative correlation between the duration of HU use with D-dimer (*r* = −0.499, *p*=0.001) and TAT (*r* = −0.401, *p*=0.010) (Figures 3(a) and 3(b)). These correlations were both statistically significant. The use of HU was a statistically significant independent predictor of D-dimer and TAT (*p* < 0.05), respectively. See Tables 3 and 4.

## 4. Discussion

The activation of the coagulation pathway and inflammation are important pathobiology in the various manifestations of SCA. Therapeutics directly or indirectly modulate the coagulation system towards normal and this could potentially minimize crisis and promote wellbeing in children living with SCA. This study determined and compared the levels of D-dimer and TAT in children living with SCA who were HU experienced and HU naïve.

The median D-dimer levels of children living with SCA who were HU naïve were elevated in comparison to those on HU. The elevated D-dimer levels seen among HU naïve children could be explained by the continuous hyperactive coagulation with activation of fibrinolytic activity characterized by consumption of anticoagulant protein (C and S), elevated tissue factor (clotting factor III), activation of platelets leading to generation of fibrinolytic end product including D-dimer [22, 23]. Our finding corroborates earlier works done by Adebola et al. [18] in Abeokuta, Fakunle and Eteng [24] in Ibadan, and Ibrahim et al. [25] in Maiduguri (all in Nigeria), Hamid et al. [26], and Fadelmula and Abdalla [27] both in Sudan who evaluated and compared D-dimer levels in children living with SCA in steady-state (HU naïve) with healthy HbAA controls. A similar finding was also reported by Kusfa et al. [28] among the adult population living with SCA reaffirming the imbalance in the coagulation homeostasis that potentiates consumptive coagulability and its role in SCA pathobiology. However, Ogunro, Akanmu, and Ekwere [29] who evaluated a mixed population (children and adults with SCA) showed no statistically significant increase in the D-dimer levels of HbSS study participants in a steady state when compared with the HbAA control group. This may be alluded to the mixed population (children and adults) as well as the small sample size in the study.

The elevated TAT values in the HU naïve study participants were also in keeping with reports by Hamid et al. [26] and El-Sakhawy et al. [30] among children with SCA and other inherited haemolytic disorders. The high levels of TAT in HU naïve children found in these studies and previous research work corroborate the background imbalance in the coagulation homeostasis which enhances hypercoagulability [26]. Furthermore, Noubouossie et al. [31], using endogenous thrombin potential (ETP) levels, as indicators of thrombin production among study participants living with SCA in steady state (2–20 years) compared to healthy controls revealed higher ETP levels in those with SCA compared to healthy controls. Thus, affirming the background hypercoagulability in the chronic haemolytic state in SCA.

The significantly lower levels of D-dimer seen in HU-experienced children living with SCA could be attributed to HU's effect in modulating the coagulation state. This effect could be explained by the ripple effect on the increase in HbF level with the resultant decrease in haemolysis, reduced red cell sickling, reduced cell adhesion to vascular endothelium and nitric oxide depletion, mitigating the possibility of consumptive coagulopathy and other pathophysiological mechanisms that will potentiate sickle cell manifestations [11, 13, 23, 32]. This finding was corroborated by Fadelmula and Abdalla [27] in a study in Sudan and Brunetta et al. [33] in Brazil. However, this is in contrast with the finding by Awoda et al. [17] which demonstrated significantly higher D-dimer levels of children living with SCA on HU in Sudan. The variation in the index study compared to Awoda et al. [17] could be explained by the difference in the age range of study participants.

The level of TAT was significantly lower in HU-exposed children living with SCA. TAT is a marker of intravascular generation of thrombin which is a central protease in haemostasis and thrombosis as well as being a key modifier for inflammation [34]. HU potentiates the reduction in TAT by lowering erythrocyte-derived microparticles and attenuating thrombin generation, reducing phosphatidylserine exposure by erythrocytes, reducing adhesive properties of erythrocytes and leukocytes, endothelial activation, nitric oxide depletion, and platelet activation [22, 23]. Our finding aligns with the studies by Elalfy et al. [35] and Colella et al. [36] both in Brazil who demonstrated similar reductions in TAT levels among patients on HU. Brunetta et al. [33] likewise demonstrated similar findings in the adult population. Furthermore, Colella et al., showed that the use of HU is associated with reduced expression of tissue factor mRNA and protein expression that enhances the activation of the coagulation cascade. Thus, the lower TAT levels in children with HbSS on HU may reduce the risk of thrombotic events in these children.

The lower values of D-dimer and TAT were noted to further reduce with a longer duration of use of HU. This may be explained by the cumulative effect of HU over time with similar observation found in a study by Elsherif et al. [37] that demonstrated a significant decrease in TAT among study participants at 6 months of follow-up which was not present at 3 months of follow-up. This agrees with the report by Noubouossie et al. [31], which showed that the duration of HU treatment was an independent predictor affecting the generation of thrombin.

This study demonstrated increased Hb levels and decreased platelet counts among subjects on HU compared to HU naïve counterparts. Although there was no statistically significant difference in the haematological parameters between the two groups, the finding showed a relative effect of HU. This is in concordance with previous studies that demonstrated a statistically significant increased Hb level, decreased white blood counts (WBC), and platelet counts among those on HU [7, 38–40]. The increased Hb level among HU-exposed children affirms previous explanations of the effect of HU on erythrocytes through increased mean corpuscular Hb, improved cellular hydration, reduced red cell deformability, reduced haemolysis and few sickled Hb cells [22, 41–43]. The improved Hb levels relatively have been explained to inhibit sickle red cell-mediated coagulation hyperactivation and endothelial dysfunction that could potentiate vascular damage, inflammation, and thrombosis resulting in various forms of sickle cell crisis [44, 45]. The decreased platelet counts and WBC could be explained by the effect of HU on the reduction of bone marrow production of neutrophils and platelet counts. Thus, altering the degree of inflammation potentials, reducing the risk of cellular adhesions, hypercoagulability, and the various crisis and end-organ damage [22, 42, 44]. The cumulative effects of HU on the cascade of events could help potentiate the salutary benefits on health and well-being in children and adults living with SCA. While reduced WBC and platelet counts are desirable, there is a need for continuous monitoring of the haematological profile (white cell count and platelet counts) on best practices to avert the possible complications of infections and bleeding disorders among individuals living with SCA [42, 45, 46].

## 5. Conclusion

Our study revealed that the levels of D-dimers and TAT are significantly lower in children living with SCA on HU when compared with HU naïve children. In addition, the duration of HU use is a predictor of reduction in the coagulation profile among children living with SCA. Thus, affirming that HU use among children living with SCA has an effect in modulating the coagulation pathway evidenced by D-dimer and TAT levels among other known therapeutic effects on the blood rheology and resultant improved health of SCA population. Therefore, to optimize the health of children living with SCA, clinicians should continue to advocate and recommend the routine use of HU in their care, with routine monitoring of the coagulation profile and haematological parameters, alongside clinical assessment for risk stratifications and management to ensure maximal health impact.

### 5.1. Strengths and Limitation

The assessment of the coagulation profile for the study participants before the commencement of the HU was not done as this was a cross-sectional study. This could have afforded us the baseline values, and a follow-up could have been done for a better understanding of the degree of change in the coagulation profile. Another limitation of the current study was that adherence to HU therapy was not assessed in the patients included in the treatment arm. The evaluation of the association between the degree of HU adherence and the coagulation profile in the population will provide a robust in-depth view of HU's effect on the coagulation profile. However, the study provides further insights into the mechanism of HU in attenuating the coagulation cascade in children living with SCA.

## Figures and Tables

**Table 1:** Sociodemographic characteristics of participants.

Characteristics	Hydroxyurea use (*n* = 40) *n* (%)	Hydroxyurea naïve (*n* = 40) *n* (%)	Total	*χ* ^2^	*p* value
Age group (years)					
2–5	7 (17.5)	7 (17.5)	14 (17.5)	0.000	1.000
6–10	8 (20.0)	8 (20.0)	16 (20.0)		
11–14	12 (30.0)	12 (30.0)	24 (30.0)		
15–18	13 (32.5)	13 (32.5)	26 (32.5)		
Mean ± SD	11.35 ± 4.6	11.35 ± 4.6		0.000^b^	1.000
Gender					
Male	21 (52.5)	21 (52.5)	42 (52.5)	0.000	1.000
Female	19 (47.5)	19 (47.5)	38 (47.5)		
Social class					
Upper	9 (22.5)	5 (12.5)	14 (17.5)	1.397	0.497
Middle	19 (47.5)	21 (52.5)	40 (50.0)		
Lower	12 (30.0)	14 (35.0)	26 (32.5)		
Number of siblings with HbSS					
None	31 (77.5)	22 (55.0)	53 (66.3)	7.256	0.027^a^
One	9 (22.5)	13 (32.5)	22 (27.5)		
Two	0 (0.0)	5 (12.5)	5 (6.3)		

^a^Statistically significant.

^b^Independent Student's *t*-test.

**Table 2:** Median comparison of coagulation profile according to duration of hydroxyurea use.

Characteristics	Duration of hydroxyurea use		*U* value	*p* value
	≤ 12 months (*n* = 15) Median (Q1–Q3)	> 12 months (*n* = 25) Median (Q1–Q3)		
D-dimer	74.42 (55.5–103.1)	20.10 (15.7–25.0)	−3.172	0.002∗
TAT	88.04 (30.4–162.9)	17.90 (10.6–32.0)	−2.739	0.005∗

*Note: U* value = Mann–Whitney *U* test.

^∗^Statistically significant.

**Table 3:** Linear regression showing independent predictors of D-dimer.

	*B* value	Adjusted *B* value	95% CI	*p* value
Age	0.019	0.013	0.241, 0.244	0.990
Male gender	0.748	0.896	−15.113, 38.927	0.377
Platelet count	2.006	1.944	−0.004, 0.193	0.060
Haemoglobin level	0.143	0.159	−10.345, 12.101	0.875
Retic count	−0.429	−0.444	−3.405–2.904	0.629
WBC counts	−0.621	−0.651	−3.748, 1.921	0.517
Hydroxyurea use	0.474	0.370	49.265, 194.629	0.001∗

^∗^Statistically significant.

**Table 4:** Linear regression showing independent predictor of thrombin antithrombin complex.

	*B* value	Adjusted *B* value	95% CI	*p* value
Age	−0.037	−0.050	−1.967, 1.254	0.661
Male gender	0.043	0.006	−164.95, 174.332	0.956
Platelet count	0.117	−0.105	−0.382, 1.046	0.356
Haemoglobin level	−0.095	−0.107	−85.819, 74.573	0.889
Retic count	0.311	−0.381	−0.931,34.022	0.583
WBC count	0.105	0.146	−0.9278, 34.388	0.255
Hydroxyurea use	0.245	0.298	58.988, 394.731	0.009∗

^∗^Statistically significant.

## Data Availability

The datasets used and/or analysed during the study to support the findings of this study are included within the article and are available by the corresponding author upon reasonable request.
